# Cerebrospinal fluid concentrations of inflammatory markers in Parkinson’s disease and atypical parkinsonian disorders

**DOI:** 10.1038/s41598-018-31517-z

**Published:** 2018-09-05

**Authors:** Sara Hall, Shorena Janelidze, Yulia Surova, Håkan Widner, Henrik Zetterberg, Oskar Hansson

**Affiliations:** 1 Department of Clinical Sciences of Malmö and Lund, Lund University, Lund, Sweden; 2 Memory Clinic, Skåne University Hospital, Malmö, Sweden; 3 Neurology Clinic, Skåne University Hospital, SE-205 02 Malmö, Sweden; 4 Department of Molecular Neuroscience, UCL Institute of Neurology, Queen Square, London, UK; 5 Clinical Neurochemistry Laboratory, Sahlgrenska University Hospital, Mölndal, Sweden; 6 Institute of Neuroscience and Physiology, The Sahlgrenska Academy at University of Gothenburg, Mölndal, Sweden; 7 UK Dementia Research Institute, London, UK

## Abstract

Inflammation has been implicated in the pathogenesis of Parkinson’s disease (PD). We here investigate levels of inflammatory biomarkers in cerebrospinal fluid (CSF) in PD and atypical parkinsonian disorders (APD) compared with neurologically healthy controls. We included 131 patients with PD and 27 PD with dementia (PDD), 24 with multiple system atrophy (MSA), 14 with progressive supranuclear palsy (PSP) and 50 controls, all part of the Swedish BioFINDER study. CSF was analyzed for CRP, SAA, IL-6, IL-8, YKL-40 and MCP-1 (CCL2) as well as α-synuclein (α-syn), tau, tau phosphorylated at Thr181 (P-tau), Aβ_42_ and NfL. In this exploratory study, we found higher levels of the inflammatory biomarker SAA in PDD and MSA compared with controls and PD and higher levels of CRP in PDD and MSA compared with PD. YKL-40 was lower in PD compared with controls. There were multiple positive correlations between the inflammatory markers, α-syn and markers of neuroaxonal injury (NfL and tau). In PD, higher levels of inflammatory biomarkers correlated with worse motor function and cognitive impairment. Thus, inflammatory biomarkers were increased in PDD and MSA. Furthermore, inflammatory biomarkers correlated with more severe disease regarding motor symptoms and cognitive impairment in PD, indicating an association between inflammation and more aggressive disease course. However, the results need confirmation in follow-up studies.

## Introduction

During the last couple of decades, inflammation has gained support in the pathogenesis of Parkinson’s disease (PD)^1^. Focus has mainly been on activated microglia and astrocytes^1,2^. However, the peripheral immune system has also been implicated^3^. Furthermore, increased expression of inflammatory mediators including cytokines and chemokines have been found in post mortem studies in PD^4^.

Microglia are resident immunocompetent and phagocytic cells in the central nervous system (CNS). They can be beneficial and serve as scavengers, phagocytosing dead cells and releasing neurotrophic substances, but they can also be harmful, upregulating a variety of proinflammatory and neurotoxic substances such as reactive oxygen species and cytokines^5^. Post mortem and PET studies have also shown increased microglia in relevant structures in the brain of PD patients^2,6,7^. Evidence of activated microglia has also been found in atypical parkinsonian disorders (APD), i.e. multiple system atrophy (MSA), progressive supranuclear palsy (PSP) and corticobasal degeneration (CBD)^8–10^. The role of microglia in the onset and progression of neurodegenerative disorders such as PD remain unclear but warrants further investigations as reviewed in Gomez-Nicola and Perry^11^.

Astrocytes are immunocompetent cells that are sensitive to proinflammatory cytokines and chemokines. They are thought to be activated as a response to injury-induced cytokine release^12^, and GFAP-positive astrocytes have been found to correlate inversely with dopaminergic cell death in PD^13^. Also, α-synuclein (α-syn)-positive astrocytes have been found to correlate positively with cell death in PD^14^ suggesting that α-syn impairs astrocytic function. However, astrocytic activation may also in itself induce detrimental effects including increased neuroinflammation and neuronal damage^15^.

Levels of inflammatory markers such as cytokines in CSF have been relatively sparsely investigated in PD and APD. YKL-40, also known as chitinase-3-like-1, is a glycoprotein that is upregulated under inflammatory conditions. In the CNS, YKL-40 is mainly expressed in astrocytes and microglia^16^. A recent study found increased levels of YKL-40 in both CSF and brain tissue in patients with Alzheimer’s disease and Creutzfeldt-Jacob but unchanged in the PDD/DLB group compared with controls with neurological controls^17^. Other studies suggest that CSF YKL-40 concentrations are either decreased or unchanged in PD whilst CSF YKL-40 concentration may be increased in APD^18–20^. Also, interleukin (IL)-6, a cytokine involved in the acute response, might be increased in the CSF of de novo PD patients and inversely correlate with motor severity in PD^21^. Further, the CSF levels of CRP seem to be increased in PD patients with dementia (PDD), and correlate with depression and fatigue in patients with PD^22^. Our group has previously shown increased levels of IL-8 in PDD compared with controls^23^.

In this cross-sectional study, we investigate further the association of the levels in CSF of acute phase proteins (CRP and SAA), IL-6 and IL-8, as well as monocyte chemotactic protein-1 (MCP-1); also called chemokine (C-C motif) ligand 2, a chemokine that is associated with microglial activation; and YKL-40 in patients with PD or APD as compared to healthy controls. We selected these markers both to replicate earlier findings^18–23^ and to extend these by including the markers SAA and MCP-1. Further, we investigated if these markers correlate with other CSF biomarkers of importance in these disorders (T-tau, P-tau, Aβ_42,_ NFL and α-syn) as well as with cognition and/or motor function.

## Methods

### Participants

This study was performed at the Clinic of Neurology, Skåne University Hospital, Sweden as part of the Swedish BioFINDER Study (www.biofinder.se). The study participants are primarily recruited from the southern region of Sweden. In this study, we included 131 patients with PD and 27 patients with PDD. We also included 24 patients with MSA and 14 with PSP. Patients with PD met the NINDS Diagnostic Criteria for PD^24^. Patients with PDD met criteria for PDD at baseline^25^. Patients with MSA met the consensus statement^26^. Patients with PSP met the criteria according to the report of the National Institute of Neurological Disorders and Stroke–Society for Progressive Supranuclear Palsy International Workshop^27^. We also included 50 neurologically healthy controls. All controls underwent cognitive testing and neurologic examination by a medical doctor and individuals with objective cognitive or parkinsonian symptoms were not included.

### Clinical assessment of study participants

A thorough medical history was taken and the patients underwent extensive testing, both regarding motor symptoms and cognition. Patients were examined by a physician experienced in movement disorders and a registered research nurse using, among other scales, the Unified Parkinson’s Disease Rating Scale (UPDRS) -3 and the Hoehn & Yahr scale^28,29^. Patients were classified as PIGD or tremor-dominant^30^. Patients with MSA were also assessed using the UMSARS (United MSA Rating Scale) and patients with PSP were assessed using the PSPRS (PSP Rating Scale)^31,32^. The study participants’ cognitive function were assessed using e.g. the Mini Mental State Examination (MMSE), 10 word-list immediate and delayed recall test of the Alzheimer’s Disease Assessment Scale (ADAS), A Quick Test for Cognitive Speed (AQT), and the one minute phonetic verbal fluency test (Letter S Fluency test)^33–36^. The participants’ levels of anxiety, depression and fatigue were assessed using HADS and FACIT-f ^37,38^.

Patients were also asked for comorbidities and any symptoms of infection during the last month and any other known medical condition associated with inflammatory response such as arthritis. Use of anti-inflammatory medication was recorded.

All individuals gave written informed consent. The study procedure was approved by the local ethics committee at Lund University Sweden and conducted according to the Helsinki Declaration.

### CSF Samples

CSF samples were obtained by lumbar puncture in the L3/L4 or L4/L5 interspace with patient non-fasting, in the morning. The samples were collected in polypropylene tubes and gently mixed to avoid gradient effects. All samples were centrifuged within 30 minutes at +4 °C at 2000 g for 10 min to remove cells and debris, and then stored in aliquots at −80 °C pending biochemical analysis. The procedure followed the Alzheimer’s Association Flow Chart for CSF biomarkers^39^. CSF YKL-40 concentration was measured by solid phase sandwich ELISA according to the manufacturer’s instructions (R&D Systems, Inc., Minneapolis, MN, USA). CRP, SAA, IL-6, IL-8, and MCP-1 were analyzed using V-PLEX Custom Human Biomarkers kit (Meso Scale Discovery, Rockville, MD, USA) according to the manufacturer’s recommendations. α-syn concentration was measured using the Alpha-Synuclein ELISA Kit (Meso Scale Discovery, Rockville, MD, USA). Hb was analyzed using an assay provided by Bethyl lab. Inc. Total tau (T-tau), tau phosphorylated at Thr181 (P-tau), Aβ_42_ and NfL have previously been described^40^.

All analyses were performed using one batch of reagents by board-certified laboratory technicians who were blinded to clinical data. For α-syn, only samples with Hb < 200 ng/L was used.

### Statistics

Statistical analyses were accomplished with SPSS for Windows, version 22.0 (SPSS Inc., Chicago, Illinois). To compare demographic data between groups, we performed a Kruskal-Wallis and pairwise Mann-Whitney U-test was then used for continuous variables. For gender differences the chi-square test was used. To test for confounders, univariate associations between two continuous variables were analyzed using the Spearman’s Rho (skewed variables and/or ordinal data). Comparisons of CSF inflammatory biomarkers between the different diagnostic groups were first investigated with a Kruskal Wallis and pair-wise Mann-Whitney U-tests. For analysis correcting for confounders, univariate general linear models adjusting for age, gender, disease duration, inflammatory condition (n = 29) and total somatic illness were then used. As described in Lindqvist *et al*.^22^ a composite score for total somatic illness was created for the most common comorbidities other than the presence of inflammatory condition (chronic inflammatory disease, acute infection and/or the use of anti-inflammatory medication) which was used as a separate covariate. Due to non-normally distributed data, ln-transformed variables were used for the univariate general linear model and regression analysis. In correlation analysis, all PD cases (including those with dementia) were included in one group. Correlations between ln-transformed CSF biomarkers as well as correlations between clinical test scores and ln-transformed inflammatory biomarkers were investigated with linear regression to correct for age, gender, disease duration, inflammatory condition and total somatic illness. Clinical test scores were ln-transformed in the case of non-normally distributed variables.

## Results

### Demographics

**Figure 1:**
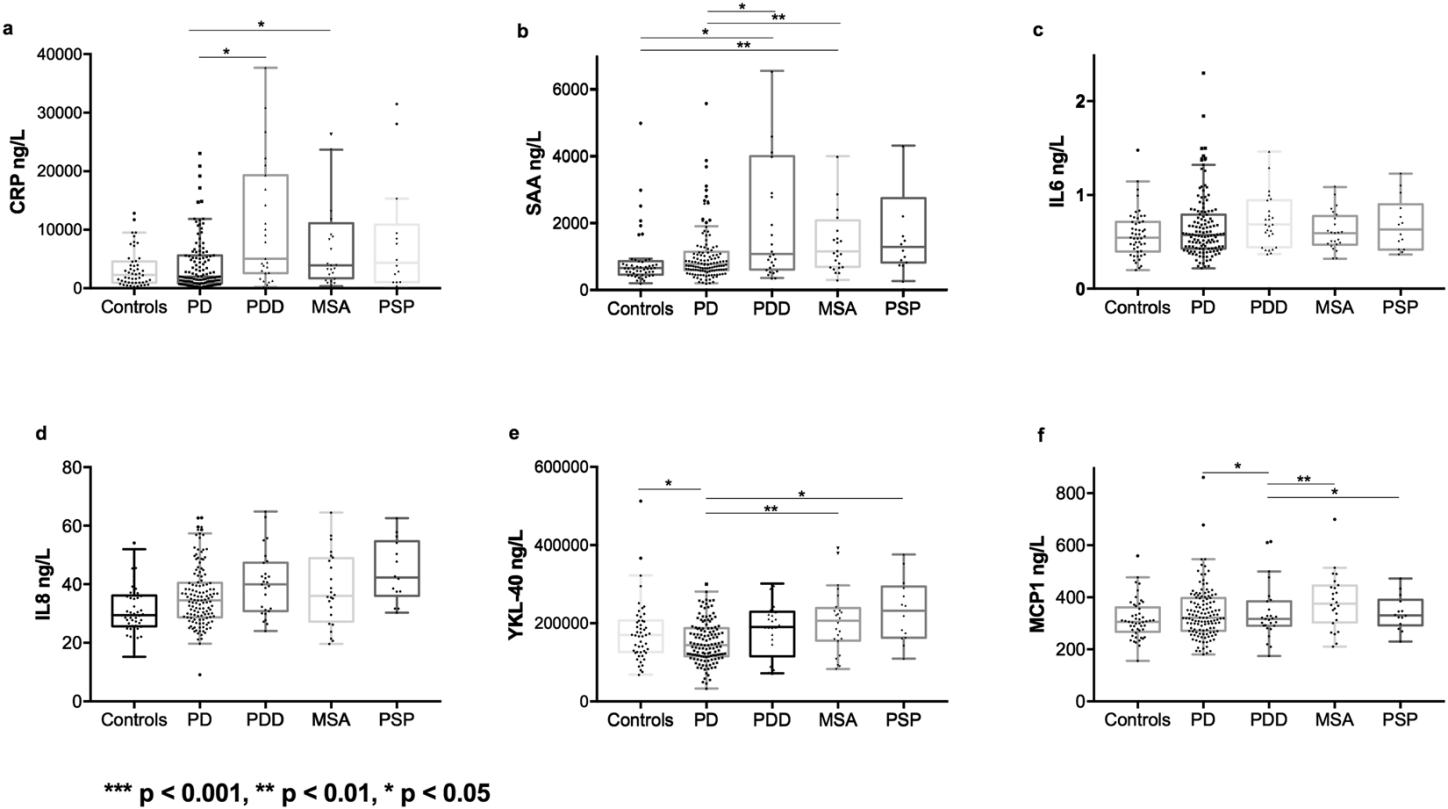
Inflammatory CSF biomarkers in different diagnostic groups. Box plots with scatter with levels of inflammatory CSF biomarkers (**a**) CRP, (**b**) SAA, (**c**) IL-6, (**d**) IL-8, (**e**) YKL-40 and (**f**) MCP-1 in the different diagnostic groups presented as median and inter quartile range. Outer whiskers are 1.5 IQR. P values are from univariate general linear model adjusting for age, gender, disease duration, inflammatory condition and total somatic illness. In figure **a**, there were 4 outliers in PD and 2 in MSA outside the axis limit. In figure **b**, there were 5 outliers in PD, 3 in PDD, 2 in PSP and 2 in MSA outside the axis limit. In figure **c** there was 1 outlier in PDD outside the axis limit. Outliers outside the graph limits are included in all statistical analysis.

Demographics, clinical characteristics and levels of CSF biomarkers of study participants are given in Table 1, Fig. 1 and Table 2. Men had higher levels of MCP-1 compared with women (p < 0.001), but no other inflammatory markers were associated with gender. Adding gender to the univariate general linear model and regression models with MCP-1 did not change the results. There were significant correlations between age and CRP, SAA, IL-8, YKL-40 and MCP-1 (p < 0.001– 0.004, Rs = 0.186–0.380). Disease duration correlated with IL-6 and IL-8 in the MSA group but not in any of the other diagnostic groups (p = 0.035, R_s_ = 0.431 and p = 0.010, R_s_ = 0.517, respectively). Disease duration also correlated with CRP in PDD (p = 0.034, R_s_ = 0.417) but not in PD or when analyzing PD and PDD together. Correcting for age, the correlation between disease duration and CRP remain in PDD (p = 0.045, β = 0.413). In MSA; SAA as well as IL-6 and IL-8 correlated with disease duration (p = 0.050, β = 0.418; p = 0.050, β = 0.424 and p = 0.027, β = 0.474, respectively). However, in MSA, we also found a negative correlation between MCP1 and disease duration (p = 0.008, β = −0.4638). Total somatic illness correlated with CRP (p = 0.031, Rs = 0.138). This correlation however was not seen in any individual diagnostic group. In the PD group, total somatic illness correlated only with YKL-40 (p = 0.041, Rs = 0.179).

**Table 1:** Demographics.

	Control (n = 50)	PD (n = 131)	PDD (n = 27)	MSA (n = 24)	PSP (n = 14)
Age	65.3 (8.6)	64.9 (10.6)	72.3 (5.9)^a,d^	63.8 (8.0)^g^	71.5 (6.2)^c,f,j^
Number (female %)	50 (56%)	131 (39%)^c^	27 (26%)^c^	24 (50%)	14 (64%)^i^
TD/PIGD dominant	NA	60/60, 1 UD	5/20^f^, 2 UD	NA	NA
LEDD	NA	520.1 (422.2)	892.3 (563.6)^d^	633.8 (567.2)	523.4 (297.9)^i^
Disease duration (years)	NA	5.5 (4.8)	14.2 (6.6)^d^	7.2 (4.5)^f,g^	5.7 (2.3)^g^
Hoehn & Yahr score	NA	2.0 (0.8)	3.0 (0.8)^d^	4.1 (0.9)^d,g^	4.1 (0.6)^d,g^
UPDRS-3 score	1.5 (2.5)	17.1 (10.5)^a^	35.4 (14.4)^a,d^	45.1 (19.2)^a,d,i^	45.4 (14.2)^a,d,i^
MMSE	27.8 (4.3)	28.4 (1.6)	22.1 (6.0)^a,d^	27.7 (2.4)^g^	23.7 (7.9)^b,d,k^
Letter S Fluency	17.4 (6.0)	14.5 (5.8)^b^	7.7 (4.1)^a,d^	12.9 (6.1)^b,h^	8.5 (5.4)^a,e^
ADAS Delayed Recall	2.3 (1.8)	3.2 (2.3)^c^	6.1 (2.8)^a,d^	3.2 (2.3)^g^	4.6 (2.6)^b,f^
AQT*	62.5 (14.1)	73.8 (28.8)^b^	173.9 (98.0)^a,d^	93.2 (63.0)^b,g^	188.0 (118.4)^a,d,j^
FACIT-f	10.1 (6.2)	14.9 (8.2)^a^	24.1 (11.9)^a,d^	23.9 (8.8)^a,s^	24.4 (11.9)^a,f^
HADS Depression	1.7 (2.6)	3.9 (3.5)^a^	7.7 (4.1)^a,d^	7.2 (4.2)^a,d^	6.3 (3.9)^a,f^
HADS Anxiety	2.7 (4.0)	5.0 (4.2)^a^	6.6 (4.5)^a^	7.4 (4.6)^a,f^	6.9 (4.6)^b^
Total somatic illness	0.52 (0.68)	0.47 (0.68)	0.44 (0.64)	0.58 (0.72)	0.57 (0.65)

Total somatic illness is a composite score of the presence of the four most common comorbidities (cardiovascular disease, asthma/allergies, osteoarthritis and diabetes mellitus). The data are presented as the mean and SD. Significance levels were analyzed using Mann-Whitney except for gender differences where the chi-square test was used.

NA = not applicable.

UD = undetermined.

*Patients who could not perform AQT were assigned a result of 360 sec.

^a^p < 0.001 vs. controls; ^b^p < 0.01 vs. controls; ^c^p < 0.05 vs. controls.

^d^p < 0.001 vs. PD; ^e^p < 0.01 vs. PD; ^f^p < 0.05 vs. PD.

^g^p < 0.001 vs. PDD; ^h^p < 0.01 vs. PDD; ^i^p < 0.05 vs. PDD.

^j^p < 0.01 vs. MSA; ^k^p < 0.05 vs. MSA.

**Table 2:** Levels of CSF biomarkers in the different diagnostic groups.

	Control (n = 50)	PD (n = 131)	PDD (n = 27)	MSA (n = 24)	PSP (n = 14)
α-syn ng/L	152.7 (58.2)	127.4 (42.6)	143.4 (66.4)	127.3 (60.0)	115.0 (34.6)
Tau ng/L	328.9 (116.6)	257.6 (83.2)^a^	310.3 (117.8)^f^	325.1 (96.0)^d^	274.0 (76.0)
P-tau ng/L	52.2 (17.8)	42.9 (13.2)^b^	48.6 (16.9)	38.9 (15.3)^b,h^	43.3 (16.3)
Aβ_42_ ng/L	641.1 (198.4)	614.9 (158.9)	552.1 (207.3)	544.1 (176.5)^c^	487.4 (158.8)^c,e^
NfL ng/L	887.2 (724.0)	870.2 (649.1)	1515.4 (1242.4)^a,d^	3502.0 (1932.7^)a,d,g^	2617.3 (850.1)^a,d,g^
CRP ng/L	3185.4 (3121.8)	6097.2 (16120.7)	11565.1 (14188.1)^b,e^	14455.3 (28362.7)^c,f^	8409.0 (9989.1)
SAA ng/L	849.4 (818.4)	3912.0 (26810.2)^c^	6387.9 (14646.8)^b,f^	7364.0 (24468.4)^a,f^	3011.7 (4273.6^)a,f^
IL-6 ng/L	0.586 (0.252)	0.660 (0.336)	0.815 (0.542)	0.613 (0.205)	0.684 (0.280)
IL-8 ng/L	31.4 (8.7)	35.8 (10.0)^b^	39.7 (11.0)^a^	38.1 (12.3)^c^	44.4 (10.6)^a,e^
MCP-1 ng/L	316.1 (76.6)	338.6 (98.4)	340.3 (107.5)	383.1 (108.9)	340.9 (65.9)
YKL-40 ng/L	176839 (78368)	152004 (53613)	184126 (70721)^f^	204501 (82161)^e^	231208 (80495)^c,d^

The data are presented as the mean and SD. Significance levels were analyzed using Mann-Whitney.

For α-syn only samples with Hb < 200 ng/L were used.

^a^p < 0.001 vs. controls; ^b^p < 0.01 vs. controls; ^c^p < 0.05 vs. controls.

^d^p < 0.001 vs. PD; ^e^p < 0.01 vs. PD; ^f^p < 0.05 vs. PD.

^g^p < 0.001 vs. PDD; ^h^p < 0.05 vs. PDD.

### CSF inflammatory biomarker concentrations in different diagnostic groups

Figure 1 displays CSF levels of the inflammatory markers in the different diagnostic groups. CRP was higher in patients with PDD and MSA compared with non-demented PD subjects (p = 0.043 and p = 0.018 respectively). SAA was higher in PDD and MSA compared with controls (p = 0.022 and p = 0.001 respectively). Furthermore, SAA was higher in PDD and MSA compared with non-demented PD (p = 0.034 and p = 0.001, respectively).

YKL-40 concentration in CSF was lower in non-demented PD compared with controls (p = 0.029), as well as MSA and PSP (p = 0.010 and p = 0.037, respectively). The levels of MCP-1 in CSF were lower in PDD compared with PD (p = 0.026), MSA (p = 0.008) and PSP (p = 0.036).

### Correlations between CSF biomarkers

As shown in Table 3, there were extensive correlations between the different inflammatory biomarkers in the cohort as a whole (all diagnostic groups including controls). CRP and SAA correlated strongly with each other (p < 0.001, β = 0.669) in the cohort as a whole but also in each diagnostic group (p ≤ 0.014, β ≥ 0.643). Further, CRP levels correlated with IL-8 and YKL-40 in the cohort as a whole (p ≤ 0.020, β ≥ 0.143), however when looking at the individual diagnostic groups, significant correlations only remained for IL-8 in PD (p = 0.010, β = 0.214). Furthermore, SAA correlated with YKL-40, IL-6 and IL-8 in the cohort as a whole (p ≤ 0.026, β ≥ 0.139) but in the PD group SAA only correlated with IL-8 (p = 0.002, β = 0.259). The CSF levels of IL-6 correlated with IL-8 in the cohort as a whole (p < 0.001, β = 0.285), a correlation that remained in each patient diagnostic group but not controls (p ≤ 0.024, β ≥ 0.249). IL-8 correlated with YKL-40 in the cohort as a whole (p < 0.001, β = 0.253) as well as in PD and controls (p ≤ 0.020, β ≥ 0.219) whereas IL-8 only correlated with MCP-1 in the cohort as a whole (p = 0.014, β = 0.158).

**Table 3:** Correlations between CSF biomarkers.

	YKL-40	MCP-1	CRP	SAA	IL-6	IL-8	α-syn	Tau	P-tau	Aβ_42_
MCP-1	NS									
CRP	0.143*	NS								
SAA	0.153*	NS	0.669***							
IL-6	NS	NS	NS	0.139*						
IL-8	0.253***	0.158*	0.182**	0.218***	0.285***					
α-syn	0.363***	NS	NS	0.160*	NS	0.209**				
Tau	0.4135***	NS	NS	0.161*	− 0.188**	NS	0.736***			
P-tau	0.359***	NS	NS	NS	NS	0.160 *	0.789***	0.731***		
Aβ_42_	NS	NS	NS	NS	NS	NS	0.406***	NS	0.213***	
NfL	0.376***	0.218***	0.149*	0.196**	NS	0.243***	0.137*	0.367***	NS	−0.157*

Correlations between different CSF biomarkers in the cohort as a whole, corrected for age, gender, disease duration, total somatic illness and inflammatory conditions, given as β-values.

For α-syn only samples with Hb <200 ng/L was included.

*significant for <0.05. **Significant for <0.01. ***Significant for <0.001.

NS = Not Significant.

Next, we compared the CSF levels of the inflammatory markers to the CSF levels of α-syn, and the neurodegeneration markers tau, P-tau and NfL. α-syn correlated positively with SAA, IL-8 and YKL-40 in the whole group. The CSF levels of NfL correlated with CRP, SAA, IL-8, YKL-40 and MCP-1. Further, tau correlated positively with SAA, and YKL-40 but negatively with IL-6. P-tau correlated with IL-8 and YKL-40 (Table 3).

### Correlations between inflammatory markers in CSF and clinical test scores in the whole sample

Worse results on motor function as measured by Hoehn & Yahr score correlated with CRP (p < 0.001, β = 0.268), SAA (p < 0.001, β = 0.241), IL-8 (p = 0.024, β = 0.159) and YKL-40 (p = 0.020, β = 0.168). Further, very similar correlations were obtained between Parkinson’s Disease Rating Scale-3 (UPDRS-3) and CSF inflammatory markers where the UPDRS-3 score correlated with CRP (p = 0.025, β = 0.138), SAA (p < 0.001, β = 0.205), IL-8 (p = 0.032, β = 0.134) and MCP-1 (p = 0.020, β = 0.150).

Worse cognitive performance as measured by Mini Mental State Examination (MMSE) correlated with SAA (p = 0.012, β = −0.156) and IL-6 (p = 0.036, β = −0.125). Similar findings were observed between the CSF inflammatory biomarkers and cognitive speed and attention as measured with A Quick Test for Cognitive Speed (AQT) and Letter fluency (for AQT: CRP, p = 0.011, β = 0.151; SAA, p = 0.003, β = 0.181; IL-6, p = 0.041, β = 0.118; IL-8, p = 0.011, β = 0.155; and for Letter Fluency: SAA, p = 0.013, β = −0.160).

### Correlations between inflammatory markers in CSF and clinical test scores in patients with PD

**Figure 2:**
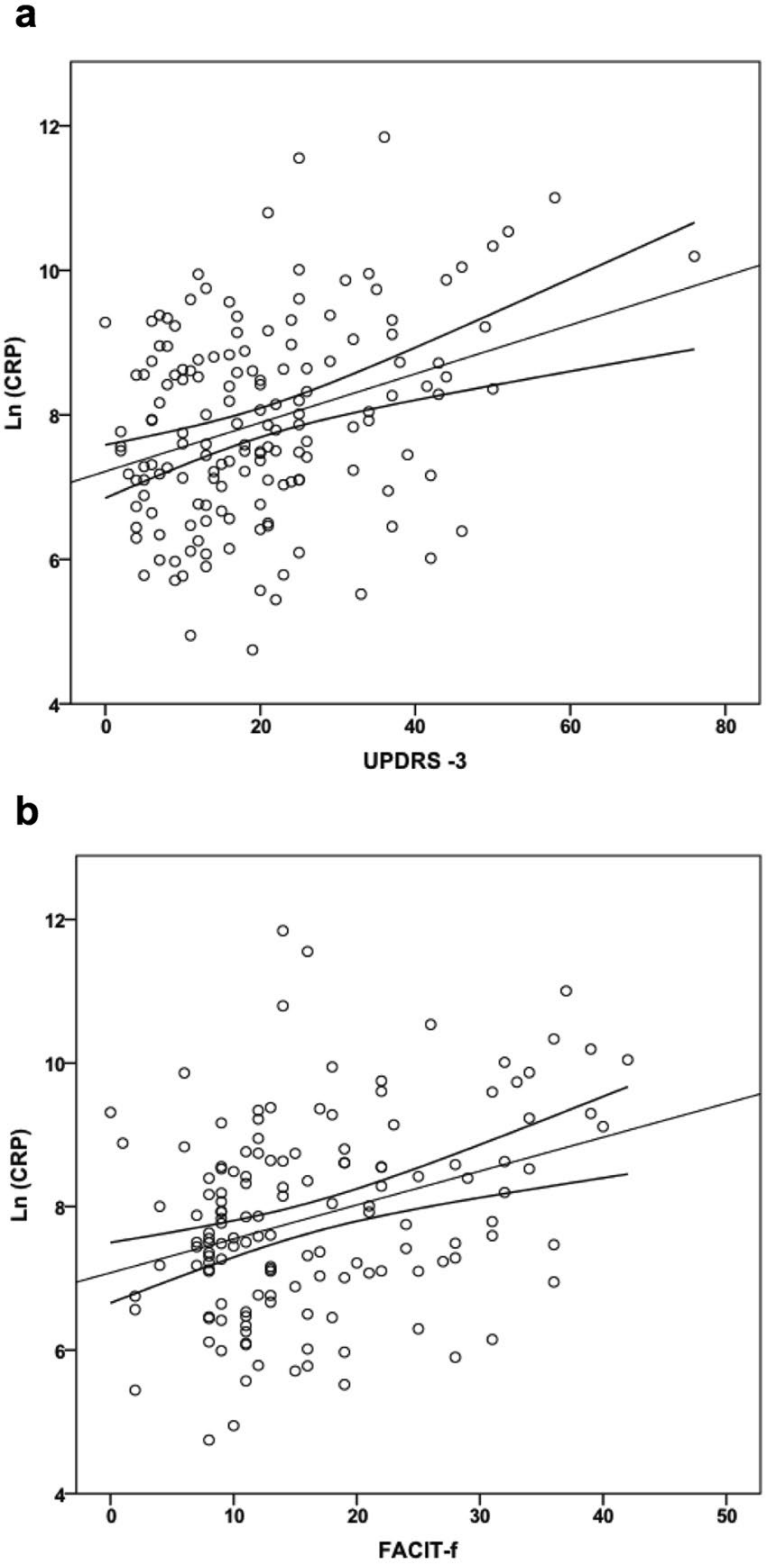
Correlations between CSF CRP and clinical test scores in PD. Correlations of ln-transformed CSF levels of CRP with UPDRS-3 (**a**) and CRP with FACIT-f (**b**) in the PD group. Lines represent linear regression and 95% CI.

In PD alone, results remained significant with correlations between inflammatory markers and motor impairment with correlations between Hoehn &Yahr and CRP (p = 0.003, β = 0.217) and SAA (p = 0.009, β = 0.192). UPDRS-3 correlated with CRP (p = 0.027, β = 0.161) and SAA (p = 0.007, β = 0.197) (Fig. 2).

Furthermore, we found significant correlations between worse results on MMSE as well as AQT and SAA (p = 0.009, β = −0.197 and p = 0.019, β = 0.172 respectively).

In PD, increased depressive symptoms as measured by the depression items of HADS and fatigue as measured by FACIT-f correlated with CRP (p = 0.006, β = 0.244 and p = 0.004, β = 0.243, respectively) and SAA (p < 0.001, β = 0.313 and p = 0.006, β = 0.235, respectively) (Fig. 2). We also observed that CRP and IL-6 correlated with lower score on HADS depression items in the control group (p = 0.009, β = −0.655 and p = 0.003, β = −0.630).

No difference in levels of CSF inflammatory biomarkers were found in tremor dominant PD compared with PIGD dominant PD.

### Correlations between inflammatory markers in CSF and motor impairment and disease stage in MSA and PSP

**Figure 3:**
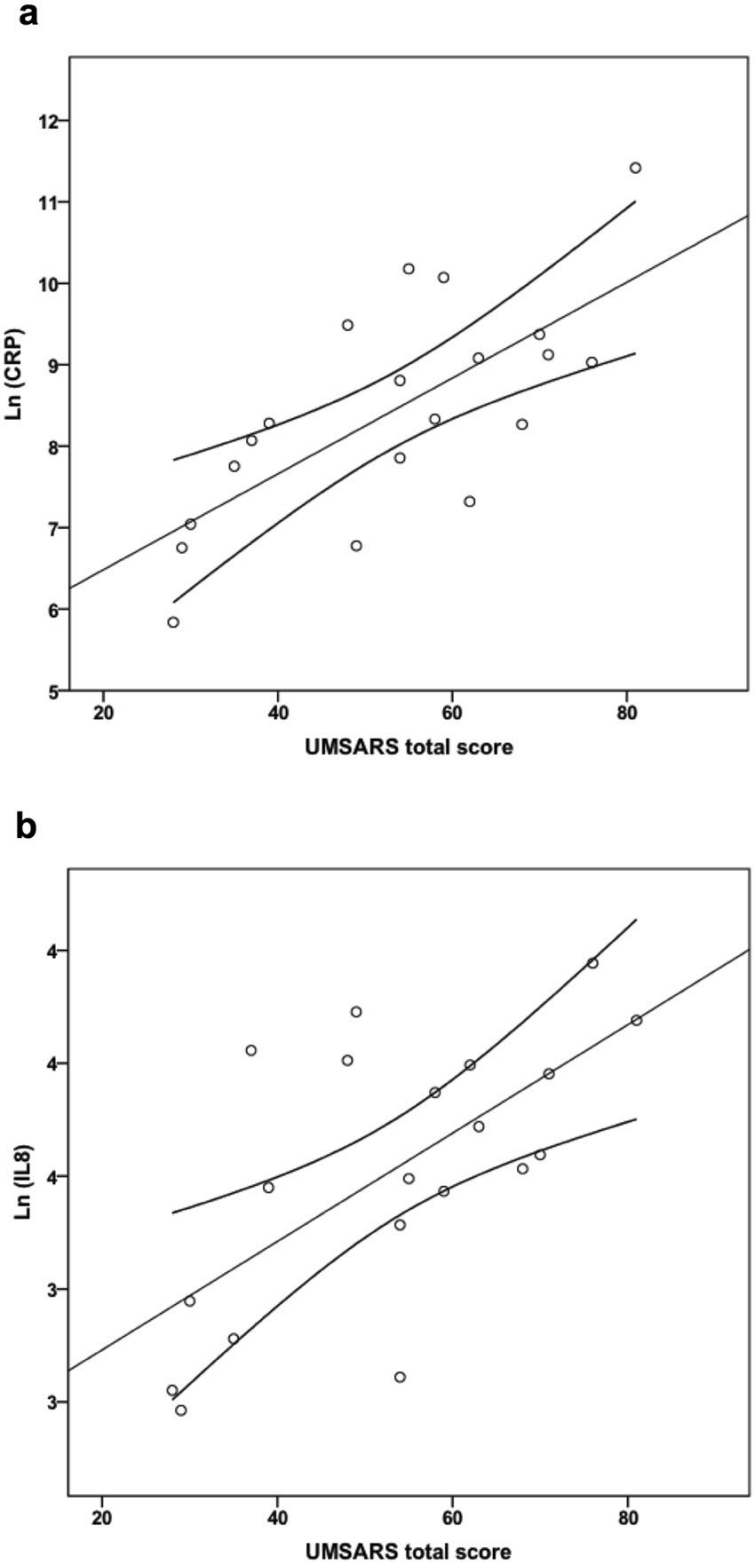
Correlations between CSF inflammation biomarkers and clinical test scores in MSA. Correlations of ln-transformed CSF levels of CRP (**a**) and IL-8 (**b**) with UMSARS total score in MSA. Lines represent linear regression and 95% CI.

In MSA, CRP and IL-8 correlated with disease severity (Hoehn & Yahr) (p = 0.038, β = 0.499 and p = 0.031, β = 0.448 respectively). Similar results were obtained using the Unified Multiple System Atrophy Rating Scale (UMSARS) total score (mean total score = 53.3, SD = 16.1) with CRP and IL-8 (p = 0.018, β = 0.618 and p = 0.039, β = 0.526 respectively) (Fig. 3). In PSP (mean total score = 45.3, SD = 14.7), IL-6 correlated with UPDRS-3 (p = 0.017, β = 0.534). No significant correlations were found between, PSP Rating Scale (PSPRS) motor score (item 3–6) or total score and inflammatory markers.

## Discussion

In this cross-sectional study, we show that the inflammatory markers CRP and SAA are higher in PDD and MSA compared with PD and in the case of SAA also compared with controls. We also find that inflammation correlates with more motor symptoms as well as cognitive decline.

Inflammation, in particular glia activation, has been linked to the pathological process in PD. In neuropathological studies, activated microglia has been found close to dopaminergic neurons in PD patients^2^ and cytokines have been found in higher levels in the striatum and substantia nigra of PD brains^7,41^. Furthermore, PET studies have shown increase microglia activation in PD compared with controls^6,42^. Microglia activation also seems to be of importance for cognitive impairment in PD. One PET study showed a larger increase in microglia activation in PDD compared with PD^43^. Further, microglia activation in PDD has been shown to correlate with decreased glucose metabolism and worse results on MMSE^43,44^. A few CSF studies further implicate inflammation in cognitive impairment in PD. In CSF, the cytokine IL-6 has been found to be increased in PD with cognitive impairment compared with non-demented PD^45^ and IL-8 been found to be increased in PDD but not PD compared with controls^23^ whereas CRP is increased in PDD compared with both PD and controls^22^. Our study can only confirm increased CRP but not IL-6 in PDD compared with PD. We also show increased levels of CRP in MSA compared with PD. Interestingly we find that SAA, another acute-phase reactant, is increased in both PDD and MSA compared with both PD and controls.

In APD, microglia activation has been found to be increased in MSA^8^ and PSP^9^. Even though traditional CSF biomarkers have been extensively investigated in APD, studies on inflammatory biomarkers in CSF in these disorders are scarce. YKL-40 has in one study been found to be increased in PSP and MSA compared with controls^20^ and another compared with PD^19^, where we now can confirm the latter.

Our findings that the inflammatory markers CRP and SAA are higher in PDD and MSA compared with PD and in the case of SAA compared with controls indicate a relationship between these disorders and inflammation. Also, high levels of YKL-40 as well as IL-8 and SAA correlate with increased CSF α-syn concentration and increased CSF concentrations of established neuronal injury markers, i.e. tau and NfL. One can speculate that α-syn pathology triggers an inflammatory response that may aggravate the disease and neuronal degeneration even further.

Our clinical data also suggest a relationship between inflammation and a more aggressive disease with correlations between inflammation and worse results on motor tests as well as cognitive impairment in PD. Further, we find that CRP correlates with disease duration in the PDD group suggesting higher inflammatory activity as time passes in this group. Interestingly, we also find that higher levels of inflammation correlate with disease duration in MSA, however, MCP1, a marker of microglia activation, correlated negatively with disease duration, suggesting a more intense microglia activation early on in the disease course.

Corroborating previous results from our group, we found a relationship between CRP and depressive symptoms as well as fatigue in PD^22^. Some of the patients were also included in the study by Lindqvist *et al*.^22^. However, in the present study we are also able to show a correlation between depressive symptoms and SAA, a marker not investigated by Lindqvist *et al*. In line with previous studies but contradicting others, we find that *lower levels* of IL-6 are associated with more depressive mood in controls^22,46^. In addition, in this study we find the same result for CRP. These results suggest that the depression may have a different pathological mechanism in PD compared with depressed non-PD patients.

Notably, PD patients had the lowest CSF concentrations of YKL-40, lower compared with both APD and controls, corroborating an earlier investigation^19^, but contrast other studies reporting unaltered CSF YKL-40 concentrations in PD^18,20^. These data may suggest that CSF YKL-40, a marker of astroglial activation, is down-regulated in PD. However, to ascertain this, in future studies, YKL-40 expression also need to be measured in brain tissue.

Astrocytes are suggested to be protective in the inflammatory response in PD and the reduction of YKL-40 may be a result of α-syn pathology, leading to defective astroglial function. On the other hand, the increase of YKL-40 in APD seem to be associated with increased release of α-syn into the CSF, or defective clearance of the protein (both potentially reflected by increased CSF α-syn concentration), and neuroaxonal injury, as reflected by the tau and NfL markers.

One major limitation of the study is the cross-sectional design making it impossible to address with any certainty weather inflammation and microglia activation is detrimental or beneficial. Furthermore, in comparisons between the diagnostic groups, significance levels were not corrected for multiple comparisons. This study should however be seen as an exploratory study. As suggested by Bender and Lange, correcting for multiple comparisons are required only for confirmatory studies, where the number of analyses are fewer. In exploratory studies, like the present one where relatively many analyses are performed, there would be a high risk of type 2 errors if correction for multiple comparisons was performed^47,48^. The present exploratory study thus needs to be confirmed in future studies.

The sample sizes in the APD groups were quite small and especially the PSP group may be underpowered. Also, there is a lack of neuropathological examination which is a limitation foremost in the APD group. However, in this study we have included well characterized patients and controls and they are followed over time to ascertain as accurate clinical diagnosis as possible. They are assessed regarding clinical characteristics making it possible to correlate the concentrations of biomarkers to both clinical status and disease severity. An added longitudinal study design may give information on the consistency of the inflammatory response and if the over-all duration of inflammatory response may correlate with disease progression and disease state. In conclusion, we show that inflammatory markers are increased in PDD and APD compared with controls and PD. We also show that this increase correlates with markers of neuroaxonal injury but also with motor impairment and cognitive decline. We show that YKL-40, an astroglial marker is decreased in PD, which may indicate impaired astrocytic function. We therefore suggest that inflammation and microglia may contribute to the disease processes in PD and APD. We believe that this study further contributes to the knowledge on the role of inflammation in PD and APD. However, further longitudinal studies in larger cohorts are needed.

## Data Availability

The datasets generated during and/or analyzed during the current study are available from the corresponding author on reasonable request when the aim is to verify the published results.
